# *In vitro* evaluation of the influence of bone cortical thickness on the primary stability of conventional- and short-sized implants

**DOI:** 10.4317/jced.58886

**Published:** 2022-02-01

**Authors:** Luiz-Antônio-Borelli Barros, Caio-Fossalussa da Silva, Germana-de Villa Camargos, Elcio Marcantonio Jr, Guilherme-José-Pimentel-Lopes de Oliveira, Luiz-Antônio-Borelli Barros-Filho

**Affiliations:** 1 DDS, pHD. Department of Diagnosis and Surgery, Univ. Est. Paul. - UNESP, Araraquara, Brazil; 2 MS. Department of Periodontology/Implantology, Dental School, Federal University of Uberlândia – UFU, Uberlândia, Brazil; 3 DDS. Department of Periodontology/Implantology, Dental School, Federal University of Uberlândia – UFU, Uberlândia, Brazil; 4 DDS. Department of Diagnosis and Surgery, Univ. Est. Paul. - UNESP, Araraquara, Brazil; 5 DDS. Department of Oral Surgery, Dental School, Universidade de Araraquara, UNIARA, Araraquara, Brazil

## Abstract

**Background:**

The aim of this *in vitro* study was to evaluate the influence of the cortical thickness on the primary stability of short and conventional-sized implants with two types of prosthetic connection.

**Material and Methods:**

Seventy-two implants were used. These implants were placed in polyurethane blocks that simulated low-density bone tissue (type IV bone), with two bone cortical heights (type I bone): 1mm and 3mm. The implants were divided into 6 groups with 12 implants each according to the type of prosthetic connections (external-hexagon -EH and morse taper- MT) and implant sizes (conventional- 4x10mm and short 5x5mm; 5.5x5mm; 5x6mm; 5.5x6mm). Insertion torque (IT) and resonance frequency analyzes (RFA) were performed to evaluate the primary stability of the implants.

**Results:**

All implants installed in blocks with 3mm of cortical thickness showed greater IT than those installed in 1mm. The short-sized MT implants had a higher IT than conventional implants of the same connection. Short-sized EH implants showed less IT than short-sized MT implants in blocks with 3mm of cortical. In blocks with 1mm of cortical, conventional EH implants had a higher IT compared to short-sized EH implants. The conventional sized implants presented higher RFA values despite the thickness of the cortical in the blocks.

**Conclusions:**

The greater bone cortical thickness and implants size provides greater primary stability of the implants regardless the prosthetic connection.

** Key words:**Implants connection, implants macrostructure, primary stability.

## Introduction

The oral rehabilitation with implants is an increasingly common practice in the field of dentistry, and the success of this type of rehabilitation depends on the success of the osseointegration process (1,2). One of the factors that must be taken into account for the osseointegration process occurs satisfactorily is to obtain good primary stability after the implant’s placement (3).

Although primary stability is obtained in the most part of native bone sites in healthy patients (2,4), in clinical conditions where implant installation occurs in areas of bone with poor density, the implant stabilization is more difficult to obtain (3,5). In addition to the reduced bone density, it is not uncommon clinical situations with reduced bone height where conventional size implants (> 10mm) cannot be installed directly in native bone (3,6).

In this context, the modifications on the implant macrostructure have been developed to facilitate the achievement of primary stability in these sites (7-9). Parameters such as the connection, thread, and platform design have been improved, making it possible to place implants in borderline conditions (5,8).

Then, the objective of this study was to evaluate the influence of implant length and prosthetic connection on the primary stability of implants placed in synthetic polyurethane blocks with poor density (mimetizing type IV bone) and different thicknesses of the high density (mimetizing the cortical bone) through the frequency of resonance and implant insertion torque.

## Material and Methods

-Experimental design

**Figure 1:**
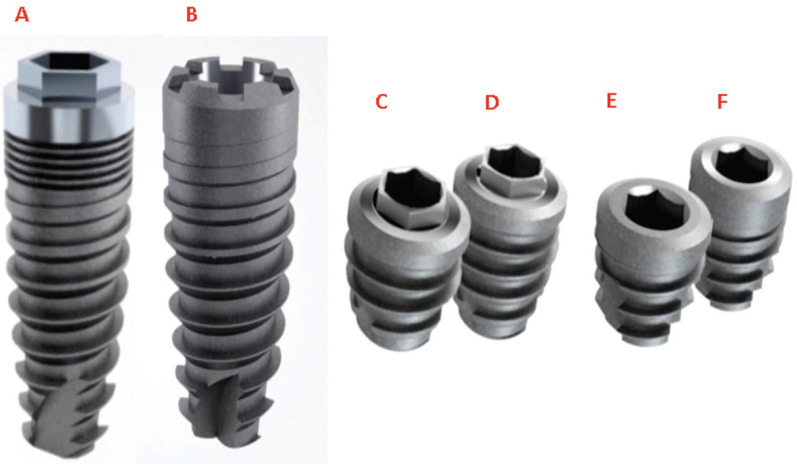
The design of the implants placed in this study. A) Conventional-sized implant with EH prosthetic connection; B) Conventional-sized implant with MT prosthetic connection; C) Short-sized implant (5 mm) with EH prosthetic connection; D) Short-sized implant (6 mm) with EH prosthetic connection; E) Short-sized implant (5 mm) with MT prosthetic connection; F) Short-sized implant (6 mm) with MT prosthetic connection. Note that conventional implants, in addition to the differences in platform type, present important differences in the region of the implant neck where the EH type implant has micro-threads and a smooth collar whereas conventional implants with MT connection have a neck without micro-threads with microroughness collar. Short implants have a similar configuration except for the type of prosthetic connection.

Seventy-two conical implants (Implacil de Bortoli, São Paulo, SP, Brazil) were used in this study. The implants presented external hexagon (EH) or morse taper (MT) connections and were allocate into 6 groups according the implants size and diameter: EH (n = 36): 4x10, 5x5 and 5x6 mm; and MT (n = 36): 4x10, 5.5x5 and 5.5x6 mm. Twelve implants from each group were installed in polyurethane blocks with Type IV density, with two bone cortical heights (1 or 3 mm) (Fig. 1).

-Implants placement

**Figure 2:**
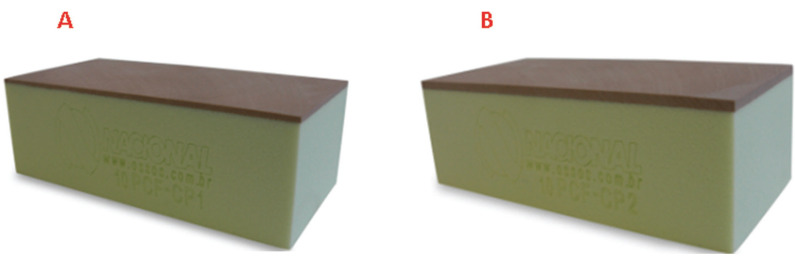
A) Specimen with 1mm of cortical thickness; B) Specimen with 3mm of cortical thickness.

The implants were inserted into the specimens by a single experienced operator, following the sequence of surgical drills recommended by the manufacturer (Table 1). In order to evaluate the primary stability of implants in conditions similar to bone tissue, specimens made of polyurethane (Nacional Óssos, Jaú-SP, Brazil) with density corresponding to type IV bone (15 PCF or 0.24 g / cm3) were used. The cortical bones had two different thicknesses (1 or 3 mm) with density similar to type I bone (40 per cubic foot (PCF) / 0.64g / cm3). The specimens presented length of 9.9 cm (L), width of 2.55 cm (L) and height of 2.02 cm (H) in order to allow the installation of 6 implants per block (Fig. 2). In total, 12 blocks were made, of which 6 had a 1mm of cortical density thickness while the other 6 presented 3mm thick of cortical density thickness.

**Table 1:**
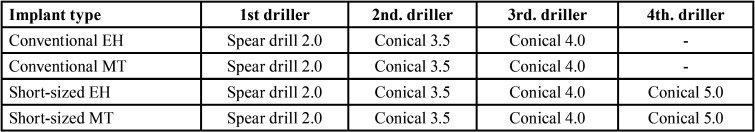
Sequence of surgical drills recommended by the manufacturer.

In order to ease the standardization during the installation of the implants into the polyurethane blocks, two surgical guides were made in colorless acrylic resin containing the same width and length of the blocks, which allowed the implants to be installed in the same position in all groups equidistant, keeping a distance of 1 cm between them and the edges of the blocks. Implants with EH type platform were installed with the platform at the level of the upper border of the blocks while the implants with MT connection were installed 2mm below the upper border of the blocks (Fig. 2).

-Evaluation of primary stability: Insertion torque and resonance frequency analysis

The primary stability of each implant was achieved through the insertion torque and the resonance frequency analysis. The insertion torque was measured in newtons (Ncm) by means of a manual torque wrench (make, model, Implacil de Bortolli, São Paulo-SP, Brazil) at the time of implants placement in the blocks until the implant was in position at bone level in the case of EH implants or two millimeters apically from the level of upper border of the blocks for MT implants. The analysis of the resonance frequency was performed with the Osstell device (Osstell - Integration Diagnostic, Göteborg, Sweden) associated with the use of a small piezoelectric transducer (SmartPegTM, Integration Diagnostics AB, Göteborg, Sweden) connected on the implant. The implant stability quotient (ISQ) measurement was performed in four faces per implant, and the ISQ of each implant value was the average of these measurements.

-Statistics

The Graphpad Prism 6 software (San Diego, CA, USA) was used to perform the statistical analyzes of this study. The insertion torque data not present normal distribution while the ISQ data presented normal distribution as detected by the Kolgomorov-Smirnov normality test. The comparison on the insertion torque of the different types of implants in each of the cortical thicknesses was performed by the Kruskall-Wallis test complemented by the Dunn test. The comparison of similar types of implants installed in blocks with different cortical sizes was performed by the Mann-Whitney test. Regarding the ISQ, the comparison on this parameter in the different types of implants in each of the cortical thicknesses was performed by the one-way Anova test complemented by the Tukey test. The comparison of similar types of implants installed in blocks with different cortical sizes was performed by the Unpaired t-test. All tests were applied with a 95% confidence level (*p* <0.05).

## Results

-Insertion torque

It was showed that greater thickness of the cortical density the greater is the implant insertion torque values, regardless of the implants size or prosthetic connection (*p* < 0.05).

Regarding the implants size, the conventional implants with EH connection (4 x 10 mm) showed higher insertion torque compared to short-sized implants with the EH connection (5 x 5 or 5 x 6 mm) regardless of the thickness of the cortical portion of the blocks (*p* < 0.05). Conversely, conventional implants with MT connection (4 x 10 mm) did not show statistically significant differences in insertion torque compared to short-sized implants (5.5 x 5 and 5.5 x 6 mm) with MT connection only for the cortical thickness of 1 mm (*p* < 0.05). In addition, short-sized implants with MT connection showed statistically higher insertion torques when compared to conventional implants with MT connection in blocks with 3 mm of cortical (*p* < 0.05).

Regarding the prosthetic connection, the conventional EH implants showed higher insertion torque compared to MT connection. In contrast, short-sized implants with MT connection presented higher insertion torque than short-sized implants with EH connection (*p* < 0.05). All the insertion torque data are showed at the Table 2.

**Table 2:**
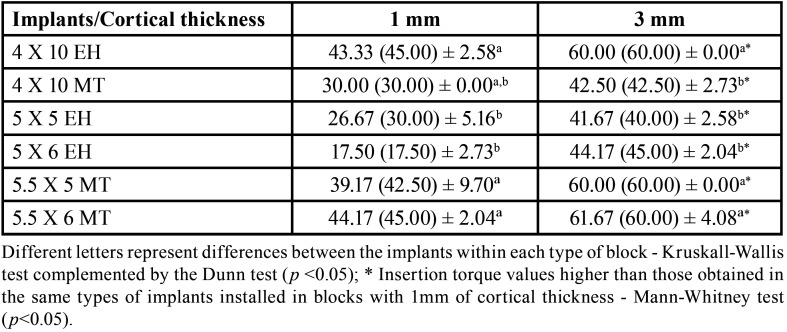
Mean (median) ± standard deviation of the insertion torque data of the implants installed in blocks with 1mm and 3mm of cortical thickness.

-Resonance frequency analysis

It was also verified that thicker cortical density is related with the higher ISQ values of the implant regardless of the size of the prosthetic connection. Groups of implants installed in a specimen with 3 mm of cortical density showed higher ISQ values than implants installed in a specimen with 1 mm of cortical density, with the exception of short implants with 5.5 x 5 mm with MT connection.

Regarding the implants size, the conventional implants showed higher ISQ values than groups of short implants, regardless of the specimen’s cortical thickness (Table 3, *p* < 0.05). Regarding the type of prosthetic connection, there was a statistically significant difference only in the ISQ values for the 5.5 x 5 mm short-sized implant with MT connection when compared to the 5 x 6 mm short sized implant with EH connection (MT: 48.42 ± 1.19 versus EH: 54.54 ± 4.61, *p* < 0.05).

**Table 3:**
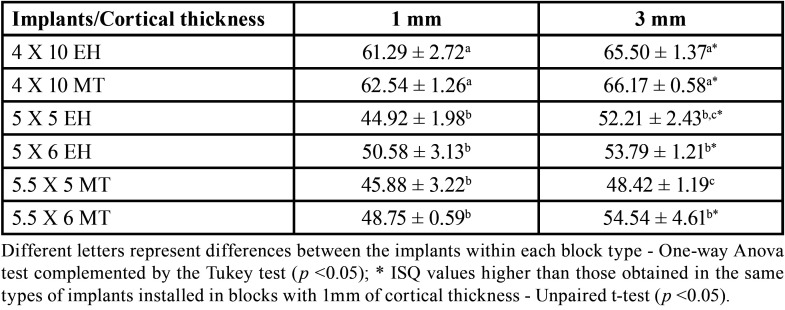
Mean ± standard deviation of resonance frequency analysis data of implants installed in blocks with 1mm and 3mm of cortical thickness.

## Discussion

This study aimed to evaluate the effect of cortical thickness on primary stability of implants with different sizes and connections using IT or ISQ. The results clearly demonstrated that dental implant stability was weakly influenced by the implants connection, but cortical thickness strongly increased implant stability.

Previous clinical studies have also found that higher cortical bone thickness increases initial implant stability, which is in agreement with the present study (10-12). Cortical bone thickness is important for the implant primary stability and occlusal loading force dissipation to the peri-implant bone tissue, whereas trabecular bone is of considerable importance for peri-implant bone healing (13,14). It can therefore be suggested that cortical bone thickness is a valuable resource to increase primary stability when planning to immediately load a dental implant (13).

In clinical situations, the obtention of a good primary stability is a fundamental requisite in order to apply the immediate loading technique. It has been showed that the IT value needed to be equal or higher than 30 Ncm (15) or the ISQ value that needed to be equal or higher than 65 (16) to provide a safe condition to indicate the immediate loading. Furthermore, it has showed that IT lower than 10 Ncm (17) and ISQ lower than 55 is related with a delayed and failures in the osseointegration process (18). In this *in vitro* study, the IT of the all implants placed in the PU with 3 mm of cortical presented mean values above 40 Ncm, while the short-sized EH implants present mean values of IT lower than 30 Ncm in the PU blocks with 1 mm of cortical. In addition, only the conventional sized implants presented ISQ higher than 60 at the both types of blocks. These findings can have important impact on clinical protocols since the immediate load can be only indicate in conventional-sized implants with safe.

Previous clinical studies have shown that short implants have success and survival rates similar to conventional implants (19). Although our findings demonstrate a disadvantage of short implants in achieving good primary stability, this does not prevent the osseointegration event from occurring successfully, despite the fact that it occurs more slowly (20). In fact, implants with reduced primary stability suck success rates, especially when maintained without prosthetic load until the establishment of secondary stability (1,18).

The MT and EH implants are indicated to be placed in different apico-coronal levels and this fact is the reason of the comparison of these different connections in this study. Then, it was observed that different prosthetic connections and the apical-coronal deepness of implants placement did not influence the primary stability of the implants. This finding is in accordance with a previous *in vitro* study that demonstrated that implants with different prosthetic connections do not present differences in their primary stability if the macrostructure was similar (21). Implants with MT connection are recommended to be inserted 2 mm below the top of the bone crest (22). This means that in clinical conditions with less cortical thickness, such as mimicked in the block with 1 mm of cortical thickness, the entire body of this implant was located into the less density portion of the PU block. It is likely that the conical macrostructure of the implants used have equated the stability of these implants installed at different levels in relation to the upper edge of the PU block with a thinner cortical.

The used to evaluate the primary stability in this study (IT or ISQ) should be interpreted independently since a high torque does not mean a high ISQ and vice versa (23). Traditionally, implant primary stability was assessed either by the clinician tactile perception or by the evaluation of IT with the help of a torque wrench or a dedicated implant motor (24). Indeed, the IT is still an easily obtainable and representative parameter for estimating the primary stability of dental implants (25). However, these methods either local objectivity or measure the rotational component of primary stability which is of lesser clinical relevance that the translational component of primary stability (21). Resonance frequency analysis (RFA) is a widely investigated objective and reliable methods of measuring translational (lateral) primary stability (18,26). RFA has become an important and widely used tool to measure the implant stability since it can assess this parameter at different time intervals in a noninvasive way, while IT can only be measured at the time of surgery (18,27,28).

This study presented some drawbacks that must be taken into account when analyzing our data. The conditions of access and ideal positioning of the implants, and the stable standard quality of the area where the implants were installed does not occur clinically, where patients have heterogeneous local conditions. Thus, this study offers only a clue of the pattern of primary stability that can occurs clinically using these types of implants. Thus, it is necessary to performs clinical evaluations in order to assess the implants tested in this study in different bone densities to better understand the effect of the design of these implants on primary stability, and consequently the influence on the osseointegration and success rates.

## Conclusions

The implant stability was weakly influenced by implant length or connection, but thicker cortical bone thickness strongly increased the implant stability.
